# Semantic Memory Structure and Self-Evaluation of Creativity: Evidence Across Tasks and Dimensions

**DOI:** 10.3390/jintelligence14030041

**Published:** 2026-03-04

**Authors:** Amit Skurnik, Yoed N. Kenett

**Affiliations:** Faculty of Data and Decision Sciences, Technion-Israel Institute of Technology, Haifa 3200003, Israel

**Keywords:** semantic memory networks, creative evaluation, metacognition, originality, usefulness

## Abstract

Creativity involves generating ideas that are both original and useful, relying on intertwined cognitive and metacognitive processes. We examined how individual differences in semantic memory structure and ideation fluency predict creative performance and self-evaluations across two studies. In Study 1, participants completed a creative problem-solving (CPS) task, with semantic memory networks estimated from a relatedness judgment task. Creative output was assessed for originality and usefulness, alongside participants’ self-evaluations. In Study 2, a within-subjects design compared participants’ output and self-evaluation of their performance in a divergent thinking task (alternative uses task) and CPS. Results revealed that ideation fluency and semantic memory network integration consistently predicted originality across tasks. In contrast, usefulness was less reliably predicted, showing task-specific associations with semantic memory network properties primarily in CPS. Importantly, self-evaluations often diverged from objective outcomes, reflecting metacognitive biases shaped by heuristic cues. These findings highlight both stable and context-sensitive mechanisms in creative performance and self-evaluation.

## 1. Introduction

Creativity is commonly defined as the capacity to generate ideas or solutions that are both original and useful (Green et al., 2024; Runco & Jaeger, 2012). It is widely regarded as an important human ability, contributing to success across academic, professional, and personal domains, and associated with various indicators of well-being (Kim, 2008; G. Scott et al., 2004). The creative process involves several interrelated stages, including the acquisition of relevant information, the formulation of ideas, the evaluation of their novelty, and the assessment of their potential usefulness (Benedek et al., 2023). These stages are supported by associative processes that rely on the organization of concepts in memory (Abraham & Bubic, 2015; Kenett, 2025), enabling individuals to identify connections between elements that may not be obviously related (Beaty & Kenett, 2023). In parallel, controlled processes facilitate an efficient search through memory, the inhibition of common responses, and the flexible adaptation of thinking strategies (Chrysikou, 2019; Volle, 2018). Executive functions such as inhibition, cognitive shifting, and broad retrieval are considered to play a central role in these processes (Benedek & Fink, 2019; Camarda et al., 2018; Krumm et al., 2018; Lebuda & Benedek, 2023).

Importantly, these cognitive mechanisms are thought to be shaped by metacognitive processes that monitor ongoing performance and regulate the allocation of cognitive resources (Lebuda & Benedek, 2023). However, scarce research has examined the role of memory in creative metacognition, especially across the various creativity dimensions (novelty and usefulness) or generalized across creativity tasks.

### 1.1. The Role of Knowledge in Creative Thinking

The generation and evaluation of creative ideas depend on a range of cognitive processes, one of which is the organization of knowledge in memory (Kenett, 2025). Semantic memory, the system responsible for storing and retrieving general knowledge about the world, encompasses not only factual information, but also the meanings of concepts and the relationships between them (Kumar, 2021). This system supports essential functions such as language comprehension, learning, and problem-solving, partly by enabling connections between concepts that may appear unrelated (Abraham & Bubic, 2015; Benedek et al., 2023). A common way to characterize its structure is through semantic memory networks (SemNets; Collins & Loftus, 1975; Siew et al., 2019). SemNets are represented via network science methodologies. Network science is based on mathematical graph theory, offering computational methods to represent complex systems, such as semantic memory, as networks where nodes represent the components of the system (e.g., words) and where edges represent the relation between nodes (e.g., semantic similarity) (Hills, 2025; Siew et al., 2019).

Extensive creativity SemNet research examines how SemNets relate to creativity as assessed by the originality of responses in tasks assessing divergent thinking. Divergent thinking (DT) involves generating multiple, varied, and original ideas from a single starting point, expanding the mental search space to explore a wide range of possibilities (Acar & Runco, 2019; Runco & Acar, 2012). A widely used DT task is the alternative uses task (AUT; Acar & Runco, 2019), in which participants generate as many alternative uses as possible for common objects such as a brick. AUT performance is typically assessed via ideation fluency (number of responses), originality (novelty and innovativeness), flexibility (diversity of conceptual categories), and elaboration (detail and complexity) (Torrance, 1966). Among these, originality, whether rated by human judges (Saretzki et al., 2025) or computational methods (Patterson et al., 2025), has been identified as a predictor of real-world creative achievement, including scientific innovation, artistic production, and entrepreneurship (Benedek et al., 2014; Jauk et al., 2014; Said-Metwaly et al., 2024).

Previous research has shown that SemNet structure can either enhance or constrain the creative process (Kenett, 2025). Several studies have applied network science methods to examine the notion that creative thinking involves the combination of distant concepts (Kenett, 2025; Kenett & Faust, 2019). Such research has found that higher creative individuals exhibit a more flexible and densely interconnected SemNet, with closely linked concepts that facilitate a wider search for ideas during the creative process (Kenett, 2025). These studies show how differences in semantic memory structure relate to individual differences in creativity, both at the group—reflecting the average SemNet of the group (Kenett et al., 2014 , 2016)—and individual—reflecting individual SemNets (Benedek et al., 2017; He et al., 2021; Ovando-Tellez et al., 2022)—levels. Across various psychometric assessments of creative thinking, more creative individuals exhibit a more connected, less structured SemNet than less creative individuals (Kenett, 2025). At the same time, a richer SemNet is not uniformly advantageous: While it may enhance ideation fluency (the ability to produce numerous ideas), it can also increase reliance on dominant, well-rehearsed associations unless balanced by cognitive control processes that promote exploration of more distant links (Kenett, 2025). Thus, while semantic memory provides the scaffolding for creativity (Abraham & Bubic, 2015; Yang et al., 2022), this role is driven by the processes that operate on it (Kenett, 2025; Volle, 2018), e.g., metacognition.

### 1.2. Metacognition and Creativity

Recent research has increasingly emphasized the central role of metacognition in creative thinking (Kenett & Ackerman, 2023; Lebuda & Benedek, 2023). Within Nelson and Narens’ (1994) framework, metacognition is typically described in terms of two complementary processes: metacognitive monitoring, which involves evaluating one’s ongoing performance, and metacognitive control, which entails adjusting strategies or effort in response to such evaluations (see Fiedler et al., 2019 for a review).

A robust body of evidence demonstrates that biases in these processes lead to discrepancies between people’s judgments and their actual performance (Erickson & Heit, 2015; Fiedler et al., 2019). These misalignments manifest as overconfidence (Dunlosky & Rawson, 2012; Ericson, 2011; Fischer et al., 2019; Kaufman et al., 2016; Metcalfe, 2013; Thaller & Brudermann, 2020), though under-confidence is also observed in some contexts (Koriat et al., 2002). One factor contributing to such biases is the reliance on heuristic cues, readily available but potentially misleading indicators, which inform judgments in domains such as memory, reasoning, and problem-solving, yet are often weakly related to actual success (Ackerman, 2019; Koriat, 1997). For example, in memory tasks, individuals may overestimate recall based on ease of processing or familiarity with the material, even when these cues do not accurately predict accuracy (Sungkhasettee et al., 2011). Similarly, in problem-solving contexts, such cues can promote premature acceptance of intuitive yet incorrect answers (Ackerman & Zalmanov, 2012; Thompson et al., 2011), and in learning environments, they may lead students to overestimate their understanding and adopt ineffective study strategies (Bjork et al., 2013; Rawson & Dunlosky, 2002). These findings indicate that metacognitive judgments, shaped by heuristics such as accessibility, prior knowledge, fluency of processing, familiarity, and distinctiveness, can systematically diverge from objective performance, a pattern also relevant to the evaluation of creativity (Ackerman & Thompson, 2017; Dodson & Schacter, 2002; Koriat, 1993; Koriat et al., 2004; Reder, 1987; Sungkhasettee et al., 2011).

Metacognitive processes are increasingly recognized as fundamental to creative performance, especially in theoretical models that highlight the iterative relationship between generating ideas and evaluating them (Finke, 1996; Kleinmintz et al., 2019; Nijstad et al., 2010; Simonton, 2023; Sowden et al., 2014). Within these frameworks, creativity is conceptualized as a dynamic cycle in which individuals continuously produce ideas and appraise them according to dimensions such as novelty and usefulness, a process that ultimately facilitates innovation (Benedek et al., 2023). Extending this view, Lebuda and Benedek (2023) argue that metacognition serves a regulatory function in creativity by directing the retrieval of relevant knowledge, assessing the quality of emerging ideas, and implementing strategic modifications when needed. This reciprocal interaction between cognitive operations and metacognitive oversight enables individuals to flexibly shift between divergent thinking, seeking a broad range of possibilities, and convergent thinking, narrowing in on the most promising solutions, depending on the demands of the task.

### 1.3. Metacognitive Evaluations in Creative Thinking

People frequently struggle to judge the quality of their own creative output accurately (Kenett et al., 2023; Sidi et al., 2020; Skurnik et al., 2025). Similar to findings in other domains of self-evaluation, these judgments are often biased or systematically distorted (Kruger & Dunning, 1999; Metcalfe, 2013). The precision of such self-assessments appears to depend on individual characteristics: individuals who are more creative or intelligent tend to show greater accuracy in evaluating their creative work (Benedek et al., 2013; Kaufman et al., 2016; Pesout & Nietfeld, 2021). In addition, situational factors, including the way tasks are structured, can shape people’s initial confidence and expectations, which in turn may anchor their evaluations or foster fixations that ultimately influence performance outcomes (Chrysikou et al., 2016; George et al., 2023).

Such effects are not incidental but reflect the fundamental nature of creativity, where ambiguity and open-endedness make accurate self-regulation particularly difficult (Silvia, 2008). This ambiguity complicates the process, as individuals must not only generate ideas but also form subjective judgments about their novelty and usefulness. These challenges underscore the difficulty of accurately self-assessing creativity and allocating effort accordingly, a crucial issue given the central role of metacognitive monitoring and control in shaping creative outcomes (Benedek et al., 2023; Lebuda & Benedek, 2023; Silvia, 2008).

### 1.4. The Relationship Between Semantic Memory and Metacognitive Evaluation

A growing line of work has documented a dissociation between objective measures of originality and people’s subjective judgments of their own originality. For example, Kenett et al. (2023) showed that, while greater semantic distance was a reliable predictor of higher originality scores, it had little impact on the subjective self-evaluation of originality, which was instead guided by heuristic cues such as the order in which ideas were produced. This pattern points to a persistent mismatch between the factors that determine originality scores and those that inform metacognitive appraisal. Such discrepancies become especially important when considering individual differences in semantic memory structure. Some individuals possess a more integrated SemNet structure, whereas others rely on more compartmentalized SemNets (Kenett, 2025). These structural differences affect the accessibility of distant associations and thus objective creative performance (e.g., Beaty et al., 2023). Yet self-judgments of originality may fail to capture these distinctions, as people often base their evaluations on a range of heuristics, including general self-perceptions of ability, situational elements like time constraints, and item-specific cues such as object familiarity, similar to processes observed in broader reasoning contexts (Ackerman, 2019).

Findings from Skurnik et al. (2025) show that individual differences in SemNet structure are associated with objective measures of originality in the AUT. These results suggest that a more integrated and densely connected SemNet may facilitate the retrieval of remote associations that contribute to originality. However, it remains an open question whether similar relationships would be observed in other dimensions of creativity—i.e., usefulness—or other types of creative tasks.

### 1.5. Originality Versus Usefulness in Creative Thinking

Creative output is inherently multidimensional, with originality and usefulness forming two core, complementary dimensions that describe the creative product, i.e., a creative idea (Runco & Jaeger, 2012; Shah et al., 2003). Originality captures the novelty or unexpectedness of an idea, whereas usefulness reflects its appropriateness and practical applicability. Both are essential for determining the value of creative products, but they do not always align. For example, Orwig et al. (2025) found a modest yet consistent inverse relationship between originality and usefulness, particularly in cases where ideas scored very high on one dimension but very low on the other. This pattern does not imply a deterministic trade-off, but it suggests that evaluators may at times struggle to reconcile these dimensions when making judgments about creative output.

Moreover, contextual factors appear to strongly shape which dimension is prioritized. Lloyd-Cox et al. (2022) demonstrated that in abstract, laboratory-based DT tasks (such as the AUT), evaluators tend to weigh originality more heavily, whereas in applied or real-world contexts, usefulness is emphasized. This context sensitivity implies that judgments of creativity are not based solely on the intrinsic qualities of the ideas themselves but are also shaped by the evaluative frame, task demands, and perceived goals of the setting.

Importantly, most of these findings are based on external raters’ evaluations, leaving open a critical question for metacognitive research: how individuals themselves balance originality and usefulness when judging their own ideas. Given that self-assessments are often guided by heuristic cues and may diverge from objective indicators, understanding how people negotiate these two dimensions in self-evaluation is essential for a fuller account of metacognition in creativity.

### 1.6. The Current Study

The aim of the current study is to examine the general role of knowledge structure in creativity across creativity tasks and dimensions. Study 1 aims to extend the knowledge regarding the relationship between SemNet structure and creative performance by addressing both key dimensions of creative ideas: originality and usefulness. Specifically, Study 1 examines idea evaluation via an ecologically valid framework by using the creative problem solving task (CPS; Reiter-Palmon et al., 1997, 1998, 2009). The CPS task requires participants to generate and assess multiple solutions to realistic, socially relevant problems. This approach allows us to investigate how participants evaluate both the originality and usefulness of their own ideas. Study 2 aims to replicate and extend Study 1. This will be achieved via a within-subjects design that compares objective performance and self-evaluation across different creative tasks (CPS vs. AUT) and dimensions (originality vs. usefulness). This approach provides a general investigation of how cognitive and metacognitive processes operate across contexts. Thus, the present study addresses a key gap in the literature by examining how semantic memory structure relates not only to creative performance, but also to self-evaluation, across both originality and usefulness and across multiple creativity tasks.

## 2. Study 1

Study 1 examines whether the relationship between SemNet structure, objective creative performance, and self-evaluation differs across the two key dimensions of creative ideas, originality and usefulness. Participants completed the CPS task under two separate rating conditions: in one block, they evaluated the originality of their ideas, and in another, their usefulness. This allowed us to examine how SemNet properties relate to both objective scores and self-evaluations of each dimension.

In the originality condition, we hypothesize that participants with more efficient and integrated SemNets would show higher objective originality, replicating previous research (Skurnik et al., 2025). In addition, we expect that ideation fluency will serve as a heuristic influencing subjective originality judgments (Kenett et al., 2023). Moreover, we expect a positive correlation between subjective and objective originality scores, but not a perfect alignment, due to the expected influence of biases. In the usefulness condition, we adopt a more exploratory approach, examining whether SemNet structure relates to the generation and evaluation of useful ideas.

### 2.1. Materials and Methods

#### 2.1.1. Participants

A total of 240 participants were recruited through Prolific—a common online crowdsourcing platform used in psychology to recruit participants (i.e., OConnell et al., 2025)—and compensated £6.50, in accordance with Prolific’s payment guidelines. The sample size was determined based on prior studies and was set to be approximately twice that used in comparable tasks, to accommodate the addition of two evaluation conditions (originality and usefulness) (Lloyd-Cox et al., 2022; Skurnik et al., 2025). Eligibility criteria required participants to be native English speakers, at least 18 years old, and to have a minimum approval rating of 95% on Prolific. The study protocol was approved by the Institutional Review Board of the Technion—Israel Institute of Technology. Four participants who failed the attention check were excluded from the analysis. The final sample included 123 female participants (51.3%), 111 male participants (46.3%), and 2 participants (0.8%) who preferred not to disclose their gender. The mean age of participants was 39.21 years (*SD* = 11.93 years), and the mean years of education was 15.44 years (*SD* = 4.10 years).

#### 2.1.2. Methods

##### **Creative Problem Solving (CPS)** 

Creativity was assessed using the CPS task (Luchini et al., 2025b; Reiter-Palmon et al., 1997, 1998, 2009). In the CPS task, participants are presented with realistic everyday dilemmas that require integrating task constraints and prior knowledge to generate solutions. These dillemmas often involve tensions between ethical considerations, interpersonal relationships, and practical constraints. For example, a dilemma may describe a situation in which an individual discovers that a close friend and coworker has engaged in unethical behavior at their shared workplace while also being financially and socially dependent on that coworker The task is designed to elicit viable, context-appropriate ideas rather than maximize the number of distant associations. The scenarios are intentionally familiar to enable the use of personal knowledge, and the structure reliably yields original yet high-quality responses (Luchini et al., 2025b; Reiter-Palmon et al., 1997, 1998, 2009).

To illustrate how originality and usefulness are evaluated in the CPS task, consider the following examples given to solve the abovementioned dilemma: A solution that is both original and useful would involve privately confronting the coworker and collaboratively developing a concrete plan to prevent future misconduct, thereby addressing the ethical violation while preserving the working relationship and the coworker’s employment. A solution that is original but not useful might suggest implementing an advanced technological monitoring system to automatically detect unethical behavior, an idea that is novel but impractical and poorly suited to the workplace context. In contrast, a solution that is useful but not original would be to report the coworker directly to the employer, a common and effective response that aligns with workplace norms but lacks novelty. Finally, a solution that is neither original nor useful would be to ignore the behavior altogether, allowing the misconduct to continue without addressing the underlying problem.

Participants engaged with ten open-ended dilemmas depicting common, everyday situations. For each participant, five dilemmas were randomly assigned to the originality condition and five to the usefulness condition. A subset of these dilemmas were based on validated CPS items developed in prior research (Acme, Becky, Brian, Joan, Sam), while the remaining dilemmas (Grace, Lily, Lucas, Noah, Olivia) were generated using the creative psychometric item generator framework (Laverghetta et al., 2024), which employs large language models to iteratively create and validate CPS items (see Supplementary Materials Text S1 for a description of all dilemmas). In each condition, participants generated at least one solution per CPS dilemma and provided a self-evaluation of their solution on a 1–5 scale, corresponding to either originality or usefulness. Participants were not given a time limit to generate solutions for each CPS dilemma, following prior work employing untimed formats for open-ended creativity tasks to reduce time pressure and support naturalistic responses (Kenett et al., 2023; Sidi et al., 2020; Skurnik et al., 2025). The order of the conditions (originality first vs. usefulness first) was randomized across participants to control for potential order effects. The following measures were computed based on participants’ performance in the CPS task:

*Ideation fluency.* In line with creativity research, ideation fluency was defined as the average number of solutions generated across the 5 CPS narratives in each condition (originality and usefulness).

*Objective originality* and *Objective usefulness* scores were generated using a large language model (LLM) trained on a dataset of expert-rated creative responses (Luchini et al., 2025b). This LLM was trained on data from thousands of human participants in order to predict CPS originality and quality. The authors show that their model reaches high accuracy levels in predicting CPS originality (*r* = 0.79) and quality (*r* = 0.83) scores when compared with human raters (see Luchini et al., 2025b). For each response, the model assigns an originality/usefulness score based on its novelty and uniqueness or the practicality of the solution. These scores range from 1 to 5, with higher values indicating greater originality/usefulness. These scores are averaged across all responses to compute a single originality score and a single quality score for each participant.

*Originality Judgments* and *Usefulness Judgments*. For each of the originality/usefulness rated dilemmas, participants evaluate the originality/usefulness of their own solution using a 1–5 scale, with higher scores reflecting greater perceived originality/usefulness. These ratings are averaged to form each participant’s originality/usefulness judgment score.

##### **SemNet Estimation and Analysis** 

Individual-based SemNets were estimated using the relatedness judgment task (RJT; Benedek et al., 2017). Following the procedure described in Skurnik et al. (2025), participants judged the relatedness of 190 word pairs, generated from ten AUT objects (Beaty et al., 2022), and ten close semantic associates for each of the AUT objects, identified via the SemDis platform (Beaty & Johnson, 2021) (see Supplementary Materials Table S1 for details of the words). In line with previous research (Bieth et al., 2024), we generated ten more words based on the closest associations to the target AUT object words. This material-generating procedure ensures variability in RJT performance and allows examination of the network properties of both target and non-target nodes. The order of the 190 word pairs was randomized for each participant.

Word pairs were presented at the center of the screen, accompanied by a continuous scale ranging from 1 to 100 that was displayed below them. A slider, labeled “completely unrelated” at one end and “completely related” at the other, was used for responses. The pointer was positioned at the midpoint of the scale at the start of each trial. Participants were instructed to make quick, intuitive judgments about the semantic relatedness of each pair without overanalyzing the relationships, evaluating each unique pair once. The task was self-paced, with no time limit imposed on individual judgments, and a short break was provided at the midpoint of the task. To ensure data quality, attention checks were incorporated into the task. Two attention tests, one before the break and one after, presented participants with pairs of identical words (e.g., Book–Book, Dog–Dog), where they were expected to rate the relatedness as 100. Participants who did not rate these pairs correctly were excluded from the analysis. Breaks were provided during the task, and participants could proceed by pressing ‘Next.’ 

**Figure 1:**
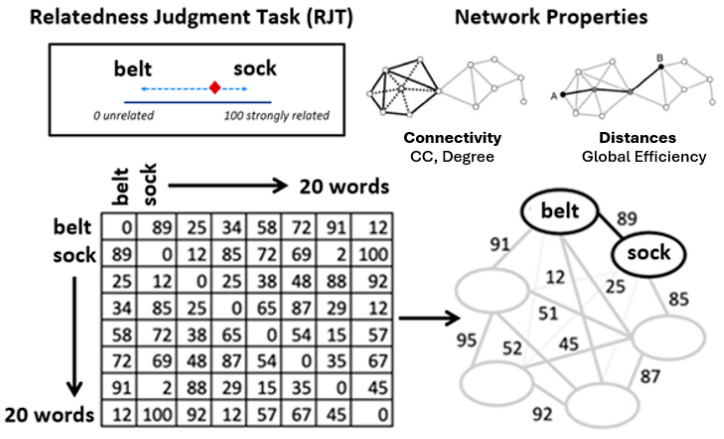
Schematic representation of the RJT and its application to estimate and analyze individual-based SemNets. Adapted from Skurnik et al. (2025).

The ratings were used to generate a symmetric 20 × 20 matrix (M), where each element (M[i][j]) represented the relatedness score between word *i* and word *j* (see Figure 1 for an illustration of the relatedness matrix and network transformation and metrics). Weighted networks were constructed using these scores as weights, with higher scores indicating stronger connections. This method enabled a detailed examination of the strength of associations between words, supporting the visualization and analysis of their semantic relationships (Benedek et al., 2017; Bieth et al., 2024).

The use of weighted (compared with unweighted, e.g., all edges having a uniform weight) networks in this study was motivated by their ability to preserve the granularity of the relatedness data, allowing for a detailed representation of semantic relationships. This approach provides a nuanced view of the structure of semantic memory, capturing variations in connection strength that are critical for interpreting complex cognitive processes. Weighted analyses are particularly suitable for small networks, as they maximize the available data and support robust and meaningful interpretations of semantic structures (Benedek et al., 2017). Furthermore, although metrics such as clustering coefficient, global efficiency, and eigenvector centrality could also be computed on unweighted networks, their weighted counterparts offer greater sensitivity to the variability in connection strength. This enables a refined characterization of an individuals’ SemNet structure; thereby shedding light on the cognitive processes these network metrics reflect.

*SemNet analysis*. Analysis was performed by computing several global and local network metrics, including the clustering coefficient, global efficiency, modularity, eigenvector centrality, and average degree. These network measures offer a comprehensive perspective on its structure and functionality (Bieth et al., 2024; Hills, 2025; Siew et al., 2019):

*Clustering coefficient (CC)* captures the degree to which nodes in the network form tightly connected groups. It represents the probability that two nodes linked to a common node are also linked to each other. Higher CC values indicate greater local interconnectivity, reflecting stronger integration of related concepts within the semantic network (Siew et al., 2019).

*Global efficiency (E-global)* reflects how effectively information can be transferred across the entire network. It is calculated as the inverse of the mean shortest path length between all node pairs, with higher values denoting a more integrated network in which concepts are easily accessible from one another (Latora & Marchiori, 2001).

*Modularity (Q)* quantifies the extent to which the network can be separated into distinct communities or clusters. A high modularity score suggests a more segmented network in which concepts are organized into clearly defined groups, whereas low modularity reflects greater interconnection between communities (Fortunato, 2010).

*Eigenvector centrality* estimates the influence of a node by considering not only its number of connections but also the connectivity of its neighbors. Nodes with high eigenvector centrality are well connected to other highly connected nodes, making them central to the overall structure of the network (Siew et al., 2019). Participant-level eigenvector centrality was calculated by averaging node scores across the network.

*Weighted average degree* represents the mean strength of a node’s connections, incorporating both the number and the weight (strength) of its links. This measure provides insight into the overall connection intensity within a participant’s network and was computed by averaging values across all nodes.

Importantly, all network metrics were computed at the individual level, yielding a single summary value for each participant’s SemNet.

#### 2.1.3. Procedure

Participants began the study by signing an informed consent form. They then completed the RJT and the CPS task. The order of these tasks was randomized across participants. After completing both tasks, participants provided demographic information before concluding the study. The full experimental session in Study 1 lasted approximately 66 min (*M* = 66.38 min, *SD* = 31.78; *median* = 59.3 min).

### 2.2. Results

We estimated participants individual-based SemNets, computed their network metrics, and analyzed participants performance in the CPS. Descriptive statistics (means and standard deviations) of all variables are presented in Table 1.

#### 2.2.1. Correlations of Main Study Variables

**Figure 2:**
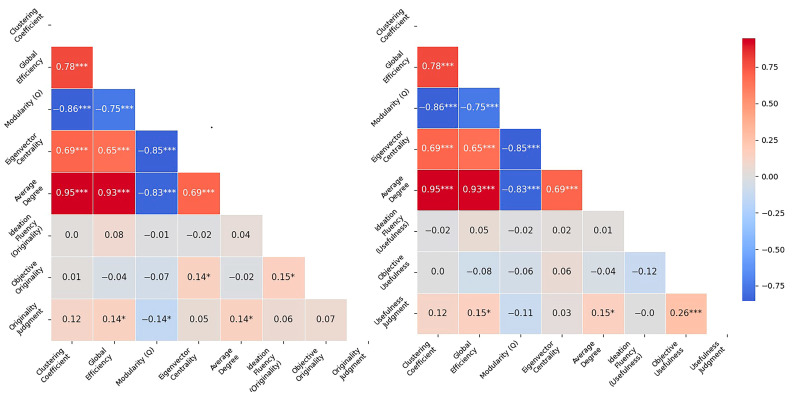
Pearson’s correlation between network metrics and creativity measures of the originality condition (**left**) and usefulness condition (**right**). The color-coded heatmap represents Pearson correlation coefficients. *—*p* < .05, ***—*p* < .001.

Next, Pearson correlation analyses were conducted to examine the associations among SemNet metrics, ideation fluency, and creativity measures, separately for the originality and usefulness rating conditions (Figure 2). Strong intercorrelations were observed among the SemNet measures. Global efficiency was positively correlated with the clustering coefficient, *r* = 0.78, *p* < .001, and negatively with modularity, *r* = −0.75, *p* < .001. Modularity was also negatively correlated with eigenvector centrality, *r* = −0.85, *p* < .001, and with average weighted degree, *r* = −0.83, *p* < .001.

In the originality condition, a significant positive correlation was found between ideation fluency and objective originality scores, *r* = 0.15, *p* = .02, indicating that participants who generated more ideas tended to achieve higher originality ratings based on the objective scoring model. Among the SemNet metrics, objective originality was positively associated with eigenvector centrality, *r* = 0.14, *p* = .019, suggesting that individuals whose networks featured more influential nodes produced more original solutions.

With respect to subjective originality, originality judgments were positively correlated with global efficiency, *r* = 0.14, *p* = .027, and weighted average degree, *r* = 0.14, *p* = .029, and negatively correlated with modularity, *r* = −0.14, *p* = .031. These results indicate that participants with more globally integrated and densely connected SemNets perceived their ideas as more original. No significant correlation was found between objective originality and originality judgments, *r* = 0.07, *p* = .29, suggesting a dissociation between external evaluations and participants’ self-assessments of originality in this condition.

In the usefulness condition, SemNet metrics showed similar intercorrelations as in the originality condition. However, the associations between network properties and creativity measures differed. In contrast to objective usefulness scores, subjective usefulness judgments were positively correlated with global efficiency, *r* = 0.15, *p* = .022, and weighted average degree, *r* = 0.15, *p* = .025, echoing the pattern observed in the originality condition. These findings suggest that more globally integrated and densely connected networks are associated with higher perceived usefulness of participants’ own responses. Finally, a modest but significant correlation was observed between usefulness judgments and objective usefulness, *r* = 0.26, *p* < .001, indicating partial alignment between self-assessed and externally evaluated creativity.

Ideation fluency was not significantly correlated with either objective usefulness, *r* = −0.12, *p* = .08, or usefulness judgments, *r* = −0.0001, *p* = .99, indicating that the number of solutions generated was not associated with either objective or self-perceived usefulness. Similarly, objective usefulness scores were not significantly correlated with any of the SemNet measures, suggesting that there was no link between SemNet structure and externally rated usefulness in this condition.

Beyond the within-condition analyses, correlations between originality and usefulness scores were also examined across conditions. A strong positive correlation was found between participants’ subjective originality and usefulness judgments, *r* = 0.76, *p* < .001, suggesting that participants who rated their ideas as more original also tended to rate them as more useful. In addition, objective originality and usefulness judgment scores were moderately correlated, *r* = 0.41, *p* < .001, indicating a partial overlap in how ideas were judged in terms of originality and usefulness by external raters.

#### 2.2.2. Regression-Based Prediction of Creativity Outcomes

To investigate the cognitive predictors of creative performance and self-evaluations, we conducted linear regression analyses separately for the originality and usefulness conditions. For the originality condition, we specified two theory-driven regression models, one predicting objective performance (originality scores) and one predicting subjective self-assessments (originality judgments). Predictor selection was guided by the results of Skurnik et al. (2025), alongside multicollinearity diagnostics aimed at improving model stability and interpretability. To this end, we excluded highly collinear variables (e.g., CC) and retained distinct network measures and fluency scores in each model. In the usefulness condition, we used an exploratory stepwise regression approach to identify key predictors, as no prior hypotheses were available. This approach complemented the hypothesis-driven analysis in the originality condition while maintaining a consistent analytic framework. Full model results are presented in Table 2.

*Predicting Objective Originality*. A regression model predicting objective originality was conducted with weighted average degree and ideation fluency as predictors. The model was not significant, *F*(2, 233) = 2.83, *p* = .06, with an adjusted R^2^ of 0.015. Among the predictors, ideation fluency significantly and positively predicted originality scores, *b* = 0.03, *SE* = 0.01, *p* = .01, while weighted average degree was not a significant predictor, *b* = −0.001, *SE* = 0.002, *p* = .65.

*Predicting Originality Judgements.* A separate model predicted subjective originality judgments with global efficiency, eigenvector centrality, and ideation fluency as predictors. The overall model was not significant, *F*(3, 232) = 2.08, *p* = .103, with an adjusted R^2^ of 0.013. Among the predictors, only global efficiency was a significant positive predictor, *b* = 0.012, *SE* = 0.005, *p* = .03, while the other variables did not reach significance.

*Predicting Objective Usefulness*. For the usefulness condition, a stepwise regression was used to predict objective usefulness scores, with global efficiency, modularity, and ideation fluency as predictors. The overall model was significant, *F*(3, 232) = 3.92, *p* = .009, with an adjusted R^2^ of 0.036. Among the predictors, global efficiency, *b* = −0.006, *SE* = 0.002, *p* = .007; and modularity, *b* = −0.51, *SE* = 0.19, *p* = .009, were significant negative predictors. Ideation fluency did not significantly predict usefulness, *b* = −0.023, *SE* = 0.013, *p* = .084.

*Predicting Usefulness Judgements*. A second stepwise regression model was conducted to predict usefulness judgments. with The eigenvector centrality and weighted average degree as predictors. The overall model was significant, *F*(2, 233) = 3.82, *p* = .023, with an *adjusted R*^2^ of 0.023. Weighted average degree was a significant positive predictor, *b* = 0.017, *SE* = 0.006, *p* = .007, whereas eigenvector centrality was not significant, *b* = −5.16, *SE* = 3.22, *p* = .111. These findings suggest that participants whose SemNets had more densely weighted connections perceived their responses as more useful.

### 2.3. Discussion

Previous research has demonstrated that individual differences in semantic memory structure are linked to objective originality in DT tasks, particularly in the AUT (Kenett, 2025), and that subjective originality judgments often diverge from objective performance, reflecting distinct underlying processes (Skurnik et al., 2025). However, these findings have been limited to a single dimension of creativity, originality, and to a specific in-lab assessment of creativity, the AUT, that exhibits limited ecological validity (Said-Metwaly et al., 2024). Study 1 extends this line of work by examining both originality and usefulness within a more ecologically valid framework, the CPS task. This approach enabled us to test whether previously observed relationships between semantic memory structure and originality generalize to a goal-oriented problem-solving context, and whether similar associations emerge for usefulness.

Our findings reveal that in the CPS task, SemNet structure did not significantly predict objective originality. Instead, ideation fluency emerged as the only significant predictor, suggesting that in more structured, goal-driven contexts, the number of ideas generated may be a stronger determinant of external originality ratings than network-level semantic properties. This pattern contrasts with prior findings in the AUT, where SemNet integration was more strongly associated with originality (Kenett, 2025), and suggests that the predictive role of semantic memory may be task-dependent.

For the dimension of usefulness, results showed a clear dissociation between external and self-assessed evaluations. Objective usefulness was not reliably predicted by SemNet metrics, whereas subjective usefulness judgments were modestly positively associated with weighted average degree. This finding indicates that individuals with more densely connected SemNets may perceive their ideas as more useful, even when this perception is not supported by external evaluations. Together, these findings point to distinct underlying mechanisms guiding internal and external assessments of usefulness.

## 3. Study 2

The findings of Study 1 highlight the need to examine whether the observed patterns hold consistently within individuals across different creativity tasks and dimensions. While Study 1 expanded prior work by incorporating both originality and usefulness in a problem-solving framework, it assessed each dimension in isolation and within a single task type. Theoretical models of creativity emphasize that idea generation and evaluation processes are dynamic (Benedek et al., 2023) and may shift depending on both task demands and evaluative criteria (Lloyd-Cox et al., 2022). A within-subject design that crosses multiple task types with both creativity dimensions can provide a stronger test of the generalizability and stability of these cognitive and metacognitive relationships. Such an approach allows us to determine whether the dissociations between objective and subjective creativity observed in Study 1 are dimension-specific, task-specific, or reflect broader individual differences in creative cognition and self-evaluation. Study 2 therefore aims to directly compare originality and usefulness across divergent thinking and problem-solving tasks within the same participants, offering a more comprehensive understanding of the mechanisms underlying creative performance and its evaluation.

Study 2 examines whether SemNet structure and metacognitive evaluations operate similarly across creative contexts. Using a within-subjects design, participants completed both the AUT and CPS task, each assessed for originality and usefulness. Furthermore, participants’ individual-based SemNets were assessed via the RJT. This approach enabled direct comparisons across tasks and dimensions, testing the generalizability of SemNet predictors and the consistency of dissociations between objective and subjective creativity, while assessing whether underlying mechanisms are task-specific or domain-general. We anticipated that the relations between semantic memory and creative performance, both objective and subjective, might vary across tasks, with different demands potentially shaping how memory organization supports or constrains creativity.

### 3.1. Materials and Methods

#### 3.1.1. Participants

A total of 120 participants were recruited via Prolific. Sample size was based on similar considerations for Study 1. Eligibility criteria were identical to Study 1. Ethical approval for the study was obtained from the Institutional Review Board of the Technion—Israel Institute of Technology. Participants were compensated £6.50 for their participation in the study. Fourteen participants were excluded from the final analysis due to failing attention checks. Therefore, the final sample consisted of 106 participants, of whom 55 were female (51.88%), 50 were male (47.16%), and 1 preferred not to say (0.94). The participants had a mean age of 38.9 years (*SD* = 13.04 years) and a mean of 16.92 years of education (*SD* = 3.79 years).

#### 3.1.2. Methods

##### **Creativity Assessment** 

To determine whether the differences observed in Study 1 compared with previous findings with the AUT (Skurnik et al., 2025) stem from task-related factors, creativity was assessed using two distinct tasks (AUT and CPS) and two evaluation dimensions (originality and usefulness). Each task included two evaluation conditions (originality and usefulness) resulting in a fully crossed within-subjects design. For each task and condition, we measured ideation fluency, objective creativity scores, and participants’ subjective self-assessments.

*Alternative Uses Task (AUT)* (Acar & Runco, 2019)—participants were presented with a total of four objects (knife, bucket, pillow, and purse). These items were selected based on their high response rates in Skurnik et al. (2025). Each participant was randomly assigned two objects to each condition, ensuring a balanced distribution across participants. The order of conditions (originality vs. usefulness) and the order of object presentation were randomized for each participant. From participants performance in the AUT, the following measures were computed:

*Ideation fluency.* Defined as the number of distinct uses per object, this was calculated as the average number of responses across the two items assigned to each condition (originality/usefulness), separately.

*Objective Originality.* To evaluate the objective originality of participants’ AUT responses, we utilized Open Creativity Scoring with Artificial Intelligence (OCSAI; Organisciak et al., 2023), an advanced AI-based platform that employs a large language model to score responses to creative tasks, such as the AUT. OCSAI was trained on thousands of AUT responses and achieves high correlation (*r* = 0.81) with human raters (Organisciak et al., 2023). Each response generated by participants was assigned an originality score by OCSAI, and these scores were averaged across all responses for each participant to calculate a mean OCSAI originality score.

*Objective Usefulness.* To evaluate the objective usefulness of participants’ AUT responses, we computed the average semantic distance using the maximum associative distance (MAD) metric and used its inverse as the usefulness score. This approach is based on findings by (Yu et al., 2025), who developed the MAD metric to measure the semantic distance between a stimulus (e.g., “pencil”) and the furthest associated word in a response, capturing response novelty and elaboration. While high MAD values indicate greater originality and semantic distance, we assume that lower MAD values reflect closer, more typical associations, therefore serving as a proxy for usefulness (see also Beaty & Johnson, 2021). In line with this assumption, the inverse of the MAD score was calculated for each response and then averaged across items to yield an objective usefulness score for each participant (Beaty & Johnson, 2021).

*Originality Judgments* and *Usefulness Judgments*—similar to Study 1.

*Creative Problem-Solving (CPS) task* (Reiter-Palmon et al., 1998, 2009), participants completed four dilemmas (Amy, Becky, Brian, and Grace; see Table S1) selected from Study 1 based on their high response rates. As in the AUT, dilemmas were randomly assigned within participants to either the originality or usefulness condition (two dilemmas per condition), and the order of conditions and dilemmas was randomized. From participants’ performance in the CPS task, the following measures were arrived at:

*Objective Originality, Objective Usefulness, Originality Judgments, and Usefulness* Judgments—similar to Study 1.

##### **SemNet Estimation and Analysis** 

Individual-based SemNet structure was estimated, and its properties were measured using the RJT, as in Study 1.

#### 3.1.3. Procedure

Participants began the study by signing an informed consent form. They then completed three tasks (AUT, RJT, and CPS) in randomized order. In both AUT and CPS, they received four items, with two assigned to originality and two to usefulness conditions. An additional attention check was included in the demographic questionnaire, and those who failed any check were excluded. The study concluded with demographic questions. The full experimental session in Study 2 lasted approximately 72 min (*M* = 71.83 min, *SD* = 31.42; *median* = 66.2 min).

### 3.2. Results

Participants individual-based SemNets were estimated, then their network properties were computed. Performance in both creativity tasks was analyzed. Descriptive statistics (means and standard deviations) of all variables are presented in Table 3.

#### 3.2.1. Correlations of Main Study Variables

**Figure 3:**
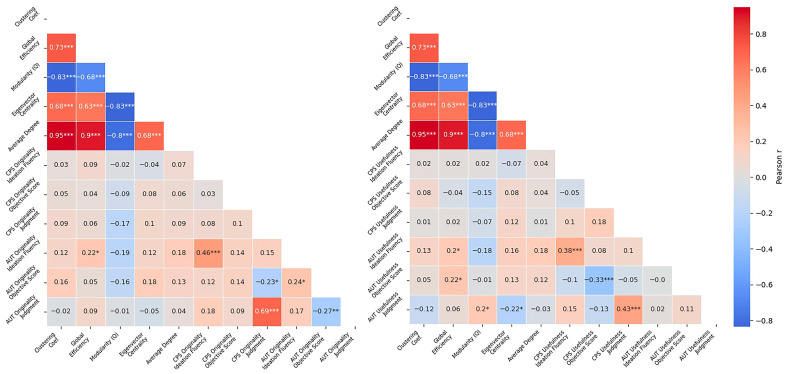
Pearson’s correlation between network metrics and creativity measures in AUT and CPS for the originality condition (**left**) and usefulness condition (**right**). The color-coded heatmap represents Pearson correlation coefficients. *—*p* < .05, **—*p* < .01, ***—*p* < .001.

To examine the relationship between participants’ semantic memory structure and their performance on the creativity tasks, we computed Pearson correlations between network-level metrics and creativity measures derived from the AUT and CPS. This was conducted separately for the originality and usefulness conditions and included both objective and subjective scores, as well as ideation fluency. Presenting these correlations side by side allows for a direct comparison across the two creativity tasks (Figure 3).

As expected, SemNet metrics were highly intercorrelated. Clustering coefficient was positively correlated with global efficiency, *r* = 0.73, *p* < .001, with eigenvector centrality, *r* = 0.68, *p* < .001, and with weighted average degree, *r* = 0.95, *p* < .001. Clustering coefficient was also negatively correlated with modularity, *r* = −0.83, *p* < .001. Global efficiency showed a significant negative correlation with modularity, *r* = −0.68, *p* < .001, and was positively correlated with both eigenvector centrality, *r* = 0.63, *p* < .001, and weighted average degree, *r* = 0.90, *p* < .001. Modularity was negatively correlated with both eigenvector centrality, *r* = −0.83, *p* < .001, and weighted average degree, *r* = −0.80, *p* < .001. Finally, eigenvector centrality and weighted average degree were positively correlated, *r* = 0.68, *p* < .001. These patterns are consistent with prior literature and indicate that the metrics capture overlapping aspects of network integration.

Most of the correlations between SemNet metrics and creativity measures across both tasks were not significant. However, global efficiency was positively correlated with ideation fluency scores in the AUT, *r* = 0.22, *p* = .026. Additionally, ideation fluency scores were positively correlated between the two tasks, *r* = 0.46, *p* < .001, indicating some consistency in generative output across divergent and problem-solving tasks.

To assess the degree of convergence between the two creativity tasks, we examined correlations between corresponding measures. A strong positive correlation was found between subjective originality judgments in the two tasks, *r* = 0.69, *p* < .001, suggesting a high degree of consistency in how participants evaluated the originality of their own ideas across task types. A smaller significant correlation was also observed between objective originality scores in CPS and AUT, *r* = 0.24, *p* < .001, indicating modest alignment in externally evaluated creative performance.

Usefulness-related measures in the CPS task, including usefulness score, usefulness judgments, and ideation fluency, did not significantaly correlate with any of the network-level metrics. In contrast, in the AUT, global efficiency was positively correlated with ideation fluency, *r* = 0.20, *p* = .040, and with usefulness scores, *r* = 0.22, *p* = .023. Subjective usefulness judgments in the AUT were positively associated with modularity, *r* = 0.20, *p* = .035, and negatively associated with eigenvector centrality, *r* = −0.22, *p* = .025. These results suggest that semantic memory structure may have a somewhat stronger influence on performance and evaluation in the AUT when participants are focused on usefulness.

Regarding convergence across tasks, ideation fluency scores in the AUT and CPS usefulness conditions were moderately correlated, *r* = 0.38, *p* < .001, as were subjective usefulness judgments, *r* = 0.43, *p* < .001. Interestingly, objective usefulness scores were negatively correlated between tasks, *r* = −0.33, *p* < .001, suggesting that participants who received higher usefulness ratings in one task tended to receive lower ratings in the other. This pattern may reflect different evaluative demands or contextual expectations embedded in the two task types.

#### 3.2.2. Task-Level Regression Predicting Creativity Scores

To investigate the extent to which semantic memory structure and ideation fluency predict creativity within each task, we conducted separate linear regression analyses for the AUT and the CPS task. For each of the four dependent variables (objective originality, subjective originality, objective usefulness, and subjective usefulness), we specified regression models based on theoretical predictions and empirical patterns observed in Study 1 and previous research (Skurnik et al., 2025).

Each model included SemNet metrics that emerged as significant or theoretically meaningful predictors in the prior studies, along with a task-specific ideational fluency score. In cases where strong correlations were observed among network metrics (e.g., between weighted average degree and clustering coefficient), we retained only one to avoid multicollinearity. Additionally, for exploratory purposes and to assess replicability, we tested the same predictor combinations from Study 1 even when they were not statistically significant in that dataset. Regression results for the AUT appear in Table 4, and those for the CPS appear in Table 5.

For the AUT, the model predicting objective originality was statistically significant, *F*(2, 103) = 3.59, *p* = .031, Adjusted R^2^ = 0.047. Among the predictors, ideation fluency significantly predicted originality scores, *b* = 0.041, *p* = .024, indicating that participants who generated more ideas tended to receive higher originality scores. Weighted average degree was not a significant predictor, *b* = 0.003, *p* = .35. In contrast, the model predicting subjective originality judgments was not significant, *F*(3, 102) = 1.74, *p* = .16, adjusted R^2^ = 0.02. The model predicting objective usefulness was significant, *F*(3, 102) = 3.24, *p* = .025, with an adjusted R^2^ of 0.06. Within the model, both global efficiency, *b* = 0.001, *p* = .002, and modularity, *b* = 0.053, *p* = .046, emerged as significant positive predictors, suggesting that more integrated and modular network structures were associated with higher usefulness ratings. Ideation fluency, however, did not predict objective usefulness, *b* = 0.0004, *p* = .72. Finally, the model predicting subjective usefulness judgments was not significant, *F*(3, 102) = 1.87, *p* = .13, adjusted R^2^ = 0.02, indicating that participants’ self-assessed usefulness was not associated with SemNet structure or fluency in this task.

In the CPS task, none of the regression models yielded statistically significant predictors. The model predicting objective originality was not significant, *F*(2, 103) = 0.23, *p* = .79, adjusted R^2^ = −0.015. Similarly, the model predicting subjective originality judgments was not significant, *F*(2, 103) = 0.61, *p* = .609, *adjusted R*^2^ = −0.01.

The model predicting objective usefulness approached significance, *F*(2, 103) = 2.28, *p* = .084, with an adjusted R^2^ = 0.036. Within the model, both global efficiency, *b* = −0.008, *p* = .045 and modularity, *b* = −0.753, *p* = .014, were significant negative predictors, replicating the pattern observed in Study 1. Ideation fluency did not significantly contribute to the model, *b* = 0.007, *p* = .65. Finally, the model predicting subjective usefulness judgments did not reach significance, *F*(2, 103) = 1.22, *p* = .299, adjusted R^2^ = 0.004.

#### 3.2.3. Combined Participant-Level Creativity Measures

To complement the task-specific analyses, we derived cross-task composite scores reflecting each participant’s general creative performance, collapsing across the AUT and CPS task. The goal of this analysis was to assess whether creativity-related individual differences (such as SemNet metrics and ideation fluency) predict creativity scores when considered beyond the specific task context.

To create cross-task composite scores for each participant, we averaged the objective originality scores and the subjective originality judgments across the AUT and CPS task. Objective usefulness was calculated by averaging the CPS usefulness score with a normalized version of the AUT usefulness score (originally based on inverse semantic distance and rescaled to a 1–5 range). Subjective usefulness reflected the mean of participants’ self-rated usefulness across both tasks. Fluency was calculated separately for originality and usefulness: for each, we summed the fluency scores from both tasks and rescaled the totals to a 1–5 scale to ensure comparability.

#### 3.2.4. Cross-Task Regression Models

The model predicting *objective originality* was significant, *F*(6, 113) = 2.65, *p* = .019, adjusted R^2^ = 0.078. Ideation fluency for originality emerged as a significant positive predictor, *β* = 0.27, *t*(113) = 3.02, *p* = .003. No other predictors reached significance. The model predicting *subjective originality* was significant, *F*(6, 113) = 2.01, *p* = .043, adjusted R^2^ = 0.043. Global efficiency significantly predicted subjective originality, *β* = 0.19, *t*(113) = 2.04, *p* = .043. The model predicting *objective usefulness* was not significant, *F*(6, 113) = 1.35, *p* = .244, adjusted R^2^ = 0.013, and none of the predictors were significant. In contrast, the model predicting *subjective usefulness* was significant, *F*(6, 113) = 2.41, *p* = .030, adjusted R^2^ = 0.068. Ideation fluency for usefulness significantly predicted subjective usefulness, *β* = 0.22, *t*(113) = 2.51, *p* = .014.

**Figure 4:**
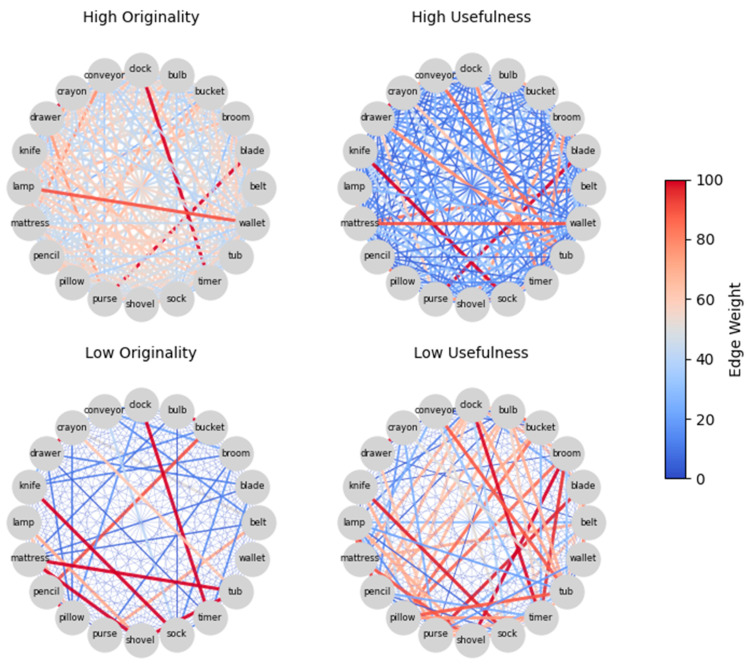
SemNets of four representative participants with high (**top**) and low (**bottom**) levels of originality (**left**) and usefulness (**right**). Edge colors and thickness represent the strength of semantic associations emphasized by warmer colors and thicker lines, and weaker associations shown by cooler colors and thinner lines.

To visually illustrate the differences in semantic memory organization associated with individual differences in originality and usefulness, we constructed SemNets for four representative participants from Study 2, based on their overall creativity scores. As shown in Figure 4, each network corresponds to a single participant: one with high originality, one with low originality, one with high usefulness, and one with low usefulness.

As can be seen, the high-originality participant’s network (top-left) appears more densely connected and exhibits greater variability in edge weights, reflecting both stronger and weaker associations across concepts. In contrast, the low-originality participant’s network (top-right) is notably sparser, with fewer and more uniformly weak connections. This visual pattern is consistent with the idea that higher originality is associated with a more richly structured and flexibly accessed SemNet. In the case of usefulness (bottom row), however, the difference between the high- and low-usefulness participant’s networks is less apparent. Both networks show a comparable number of connections and similar variability in edge weights. This lack of a clear distinction may reinforce the idea that usefulness, as compared with originality, is less strongly tied to the associative structure of semantic memory.

#### 3.2.5. Task Convergence and Internal Consistency

To examine the convergence of creativity constructs across tasks, we computed Pearson correlations between AUT and CPS scores for each variable. Subjective originality scores were highly correlated across tasks, *r* = 0.69, *p* < .001, and subjective usefulness showed a moderate correlation, *r* = 0.44, *p* < .001. Objective originality showed a marginal correlation, *r* = 0.17, *p* = .062, while objective usefulness scores were negatively correlated, *r* = −0.34, *p* < .001.

We also assessed internal consistency using Cronbach’s alpha. High reliability was found for subjective originality (α = 0.81), moderate for subjective usefulness (α = 0.61), low for objective originality (α = 0.29), and negative for objective usefulness (α = −0.13), indicating poor coherence for the latter.

### 3.3. Discussion

Study 2 extended prior research by computing combined creativity scores across tasks (AUT and CPS), evaluating convergence between tasks, and estimating the internal consistency of creativity constructs.

Ideation fluency significantly predicted objective originality in both the AUT and CPS task, with global efficiency and modularity associated with subjective originality in AUT and CPS, respectively. In contrast, neither ideational fluency nor SemNet metrics were found to be related to usefulness, whether objective or subjective. These findings, which partially replicate results from Study 1 and Skurnik et al. (2025), underscore the role of ideation fluency and SemNet connectivity in shaping both the production and perception of original ideas, while suggesting that the evaluation of usefulness may depend on distinct cognitive mechanisms not captured by the current measures.

To assess creativity beyond individual tasks, we computed composite scores across AUT and CPS. In line with the task-level results, ideation fluency remained a robust predictor of the aggregated objective originality score, and global efficiency predicted the aggregated subjective originality score. Ideation fluency also predicted aggregated subjective usefulness, whereas no variable predicted aggregated objective usefulness. Taken together, these cross-task findings reinforce the claim that originality appears to be supported by general SemNet properties and ideation fluency. This interpretation is consistent with Benedek et al. (2017), who showed that individual differences in SemNet structure, as captured through the RJT, and fluid intelligence are associated with the ability to generate original ideas. In contrast, usefulness, particularly in its objective form, may be more contextually dependent and difficult to predict from the measured variables, highlighting a potential dissociation in the cognitive underpinnings of these two dimensions of creativity.

Correlation and consistency analyses showed that subjective originality and usefulness judgments were moderately to strongly correlated across tasks, suggesting stable personal tendencies. In contrast, objective scores, especially for usefulness, were less consistent, indicating greater task dependence or measurement error. Overall, self-perceived originality may reflect a general cognitive trait, while objective usefulness appears more context specific. Finally, as in prior studies (Kenett et al., 2023; Sidi et al., 2020; Skurnik et al., 2025), Study 2 also revealed dissociations between creative performance and self-evaluation, particularly for usefulness.

## 4. General Discussion

Elucidating creative thinking requires an integrative view that encompasses both the cognitive mechanisms driving idea generation and the metacognitive processes guiding self-evaluation (Lebuda & Benedek, 2023). While previous research has made significant strides in identifying cognitive correlates of creativity, such as associative richness, semantic flexibility, and ideation fluency (Beaty & Kenett, 2023; Benedek et al., 2023; Benedek & Fink, 2019), less is known about the mechanisms by which people monitor and judge their own creative outputs (Lebuda & Benedek, 2023).

This research aimed to examine the metacognitive and cognitive underpinnings of creativity by investigating how semantic memory structure and ideation fluency predict both objective and subjective creativity outcomes across two studies. By employing SemNet analyses with ideation fluency and self-evaluation measures across multipole creativity tasks and dimensions, the work uniquely contributes to the investigation of individual differences in idea generation and evaluation.

Study 1 examined the relation of semNet structure to objective and subjective measures of originality and usefulness. Thus, Study 1 extends previous creativity SemNet research (Kenett, 2025), by assessing individual differences in creativity via the CPS. Furthermore, Study 1 relates SemNet structure to idea usefulness, and not just originality as in most previous research (Kenett, 2025). Study 2 replicates and extends Study 1, by conducting a within-subject design, relating individual-based SemNet structure to objective and subjective measures of originality and usefulness in both the AUT and CPS task. Overall, both studies delve into the role of SemNet structure across the two main dimensions of creativity—originality and usefulness (Runco & Jaeger, 2012)—and across various open-ended creativity tasks—the AUT and CPS task.

### 4.1. Ideational Fluency

Across both studies, ideation fluency consistently predicted objective originality. Generating a higher number of ideas increased the likelihood of producing novel responses, reinforcing prior findings on the role of fluent idea generation in creative performance (Bai et al., 2021; Beaty et al., 2023; Beaty & Silvia, 2012; Benedek et al., 2023). This finding supports the role of fluency as a stable cognitive predictor of originality and underscores its importance as both a generative and heuristic cue in creative evaluation.

However, in the usefulness condition no relationship between ideational fluency and either usefulness measures were found. This aligns with the notion that usefulness, unlike originality, may not be intuitively linked to idea quantity; generating more solutions increases opportunities for novelty but does not necessarily increase the likelihood of producing practical or contextually appropriate ideas (i.e., Beaty et al., 2023).

### 4.2. SemNet Structure and Originality

Our findings demonstrate how semantic memory structure further contributed to creative output. SemNet properties such as global efficiency and weighted average degree predicted higher originality scores in both the AUT and CPS task, indicating that integrated, well-connected SemNets support the generation of novel associations. Thus, our findings replicate past results regarding the role of knowledge in creativity (Kenett, 2025).

Beyond objective performance, we also examined the subjective dimension of creative evaluation, revealing that SemNet structure was associated with participants’ metacognitive judgments of their own ideas. However, these relationships were not always parallel to performance outcomes. While ideation fluency and SemNet integration consistently predicted originality, the links between SemNet structure and self-evaluations were more variable. This suggests that metacognitive judgments may sometimes rely on heuristic cues, such as ease of retrieval or ideational fluency, rather than aligning directly with objective creativity measures (Kenett et al., 2023; Sidi et al., 2020; Skurnik et al., 2025).

### 4.3. SemNet Structure and Usefulness

In contrast to the relatively consistent findings for originality, the results regarding the relation between SemNet structure and usefulness were more variable and task-dependent. Objective usefulness was only weakly predicted by SemNet metrics and was unrelated to ideation fluency in both the AUT and CPS task. This suggests that generating more ideas does not necessarily increase their practical value, and that usefulness may depend on contextual, goal-directed reasoning rather than associative semantic processes.

Notably, some SemNet metrics, such as lower modularity and higher global efficiency, were associated with greater usefulness in the CPS task, implying that more integrated knowledge structures may facilitate practical problem-solving in realistic scenarios. However, these effects did not generalize across tasks, reinforcing the idea that the cognitive mechanisms supporting usefulness are less stable and more context-sensitive than those supporting originality.

### 4.4. Generalizability Across Creativity Tasks and Theoretical Significance

Finally, we examine the generalizability of these mechanisms across creativity tasks and dimensions. While previous studies have focused on a single task or dimension (Kenett et al., 2023; Orwig et al., 2025; Reiter-Palmon et al., 2009; Saretzki et al., 2025), Study 2 incorporated a within-subjects comparison between the AUT and CPS task. By combining data from both tasks, we constructed composite indices for originality and usefulness, allowing us to assess trait-like consistency versus task-specific variability.

Our findings revealed stable individual differences in originality and its predictors—such as ideation fluency and SemNet integration—across tasks, suggesting that originality may reflect domain-general cognitive traits. In contrast, usefulness showed weaker and more inconsistent patterns, indicating stronger dependence on task context and evaluative framing. This cross-task design also enabled a comparison of metacognitive self-evaluations. While originality judgments were partially aligned with objective performance, usefulness judgments were more variable and likely influenced by contextual or interpretive factors. Overall, the results support a multidimensional view of creativity and highlight the value of studying it across diverse tasks to distinguish between general and context-specific mechanisms.

The present work offers several theoretical contributions to the study of creative cognition and metacognition. Theoretically, it extends current models of creative metacognition (Ackerman, 2019; Kenett & Ackerman, 2023; Lebuda & Benedek, 2023) by demonstrating that individual differences in semantic memory structure systematically relate not only to the generation of original ideas but also to how these ideas are evaluated. This suggests that metacognitive self-assessments are not random or detached from cognitive processes but are influenced by domain-general traits such as semantic efficiency and associative accessibility. Moreover, by explicitly incorporating both originality and usefulness, two core but often dissociated dimensions of creativity, this research underscores the need for multidimensional models that can account for how different cognitive mechanisms contribute to distinct creative outcomes.

Overall, our research bridges the gap between cognitive and metacognitive perspectives on creativity, emphasizing the need to jointly examine the relationship between knowledge structure, ideation processes, and subjective evaluation (Kenett & Ackerman, 2023). Our findings suggest that the cognitive mechanisms supporting creative evaluation may vary across dimensions and task contexts (e.g., Lloyd-Cox et al., 2022; Orwig et al., 2025). Whereas originality in open-ended tasks appears more contingent on semantic memory structure, usefulness, especially in problem-solving contexts, may be shaped by different factors, with subjective evaluations potentially influenced by internal heuristics or perceived network richness.

### 4.5. Practical Implications

In addition to their theoretical relevance, the present findings may have implications for how creative effort and resources are allocated in everyday, educational and professional contexts. The observed dissociation between objective creative performance and subjective self-evaluation suggests that individuals’ perceptions of their own originality and usefulness do not always accurately reflect their actual creative contributions (Kenett et al., 2023; Sidi et al., 2020; Skurnik et al., 2025).

Importantly, this dissociation was systematically related to individual differences in SemNet structure, which were associated with both creative performance and self-evaluative judgments across tasks. As a result, individuals may differentially invest time, attention, or effort in developing, refining, or sharing ideas based on subjective evaluations that are only weakly aligned with objective indicators of creativity. For example, individuals with more integrated SemNets—who tended to produce more original ideas—may nevertheless be less inclined to further elaborate or pursue these ideas if their self-evaluations underestimate originality.

In educational contexts, such misalignment may shape how learners engage with open-ended learning activities, persist in creative problem solving, and decide whether to revise or further elaborate their ideas. Such processes are central to deep learning and the development of creative and critical thinking skills in education. Given the close links between creativity and knowledge acquisition (Luchini et al., 2025a), systematic biases in self-evaluation may influence not only creative output but also how students regulate their learning and make use of educational opportunities (Raz & Kenett, 2026). Similarly, these findings may inform discussions in teacher training regarding individual differences in learners’ self-evaluative processes in contexts that emphasize creative and critical thinking (e.g., Goulet-Pelletier et al., 2025; Sowden et al., 2025). Consistent with this perspective, international policy frameworks have emphasized creative and critical thinking, together with metacognitive skills, as core components of contemporary education rather than peripheral outcomes (e.g., OECD, UNESCO; Barbot & Kaufman, 2025; C. L. Scott, 2023).

Finally, although the present research does not directly examine decision-making or resource allocation, our findings point to a potential link between SemNet structure, metacognitive evaluation processes, and the prioritization of creative outputs in real-world settings (i.e., Lopez-Persem et al., 2024; Moreno-Rodriguez et al., 2025).

### 4.6. Limitations and Future Directions

Despite its strengths, our research has limitations that should be addressed. This research concerns network-based predictors and both subjective and objective originality and usefulness scores aggregated across all responses of each participant. This approach provides robustness for all measures, i.e., the means as well as the within-participant correlation that underlies resolution. However, this approach does not capture object-level and trial-level processes (e.g., Silvia, 2008). Thus, our findings reflect trait-level characteristics rather than monitoring processes that might differ across task items. Moreover, although subjective evaluations were collected at the response level, the operationalization of these judgments constrained the applicability of standard trial-level metacognitive indices. Specifically, in Study 1, the use of coarse 1–5 rating scales limited within-participant variability; meanwhile, in Study 2, conceptual misalignment between subjective ratings and objective usefulness measures (e.g., inverse MAD) precluded meaningful computation of calibration and resolution indices. Relatedly, objective originality scores were derived using different LLM-based evaluation approaches across tasks (i.e., Luchini et al., 2025b; Organisciak et al., 2023; Yu et al., 2025), which may partly account for the limited convergence between measures and reflect task-specific operationalizations of originality. Future research may incorporate object-level and trial-level measures to better understand the dynamic processes underlying metacognitive evaluations in creative tasks in a more fine-grained manner. In addition, further demographic variables, such as age and education (e.g., Raz et al., 2025), should be taken into consideration in future studies to replicate and generalize our findings.

This research focused on intrapersonal metacognitive evaluations of originality and usefulness, i.e., how individuals assess the creativity of their own ideas. However, it is important to acknowledge that creative evaluation also occurs in interpersonal and social contexts (Grohman et al., 2006). While these dimensions are beyond the scope of the current study, future research could extend this line of work by investigating how semantic memory structure relates to interpersonal evaluations, or by examining how individuals assess the effectiveness of ideas in addition to their originality. Such directions would provide a more comprehensive view of creative evaluation across diverse levels of analysis.

In addition, our examination of the role of SemNet in objective and subjective usefulness was conducted via lab-based tasks, and the ecological validity of our findings call for further research. Importantly, the AUT has been shown to significantly predict real-life creativity (Said-Metwaly et al., 2024), the CPS task assesses real-life problems (Reiter-Palmon & Robinson, 2009), and SemNet structure has been shown to be related to real-life creative achievement (Ovando-Tellez et al., 2022). Future research is needed with regard to how context and domain-specificity (e.g., Lloyd-Cox et al., 2022) impact creative evaluation processes and the role that memory plays in them (e.g., Orwig et al., 2025).

Moreover, our results relating SemNet metrics and objective originality only partially replicate past research (Kenett, 2025). This may be related to the size of the estimated SemNets in our research (which are considerably smaller than past research), or low power due to the sample size (e.g., He et al., 2021; Ovando-Tellez et al., 2022). However, our sample size and network size does align with previous individual-based SemNet research (Benedek et al., 2017; Bieth et al., 2024). Thus, further research is needed to replicate and extend the role of SemNet structure in objective and subjective measures of idea originality and usefulness across creativity tasks.

Finally, the modest explained variance observed in several regression models, particularly in Study 2, likely reflects methodological constraints rather than weak underlying relationships. The within-subjects design emphasizes cross-task comparison and generalizability within individuals, thereby limiting the between-participant variance available for prediction by stable trait-level measures. In addition, creativity scores were aggregated across a small number of task instances per condition, which may increase measurement noise. Accordingly, adjusted R^2^ values should be interpreted cautiously and in light of the study’s design and aims.

## 5. Conclusions

By examining both the AUT and CPS task, we find that SemNet properties and ideation fluency consistently predict originality, while the role of semantic memory in usefulness is more variable and context dependent. Importantly, metacognitive self-evaluations did not always align with objective performance, underscoring the idea that subjective judgments of creativity may be guided by heuristic cues as much as by actual idea quality (Kenett et al., 2023; Sidi et al., 2020; Skurnik et al., 2025). Taken together, our findings highlight the value of adopting a multidimensional approach to creativity that considers both originality and usefulness, across tasks, and across levels of evaluation. Our findings further point to the need for future work to disentangle the cognitive and heuristic processes that shape how individuals generate and judge their own ideas.

Overall, our work dissociates between objective and subjective measures of creative evaluation, between the two main dimensions of creative ideation (originality and usefulness), and between different creativity tasks (AUT and CPS task). Indeed, creativity is a complex, multi-stage, multi-process, context-dependent construct. Thus, our findings shed new light on the interplay between semantic memory structure and metacognitive evaluation in creative thinking.

## Figures and Tables

**Table 1:** Descriptive statistics of all variables analyzed in Study 1.

Task	Measure	Mean	SD
Relatedness judgment task (RJT)	Clustering coefficient (CC)	0.16	0.14
	Global efficiency (E-global)	29.94	15.93
	Modularity (Q)	0.28	0.18
	Eigenvector centrality	0.21	0.02
	Weighted average degree	19.15	12.5
Creative problem solving (CPS)	Ideation fluency (originality)	2.09	2.01
	Objective originality	2.33	0.43
	Subjective originality	3.5	1.03
	Ideation fluency (usefulness)	1.96	1.82
	Objective usefulness	2.80	0.38
	Subjective usefulness	3.78	0.87

*Note*. *N* = 236.

**Table 2:** Summary of regression models results.

Outcome	F	*p*	Adj. R^2^	Predictor	Β	SE	*t*	*p*
Originality score	2.83	.06	0.015	(Intercept)	2.28	0.05	39.95	<.001
				Weighted avg. degree	−0.001	0.002	−0.44	.65
				Ideation fluency (originality)	0.03	0.01	2.35	.019
Originality judgment	2.08	.103	0.013	(Intercept)	3.73	0.67	5.57	<.001
				Global efficiency	0.012	0.005	2.19	.029
				Eigenvector centrality	−3.14	3.67	−0.86	.392
				Ideation fluency (originality)	0.02	0.03	0.69	.491
Usefulness score	3.92	.009	0.035	(Intercept)	3.17	0.11	26.75	<.001
				Global efficiency	−0.006	0.002	−2.72	.006
				Modularity	−0.51	0.19	−2.63	.009
				Ideation fluency (usefulness)	−0.02	0.01	−1.73	.083
Usefulness judgment	3.82	.023	0.023	(Intercept)	4.52	0.59	7.66	<.001
				Eigenvector centrality	−5.15	3.22	−1.6	.110
				Weighted avg. degree	0.01	0.006	2.73	.006

*Note*—Reported values include unstandardized regression coefficients (B), standard errors (SE), *t*-values, and exact *p*-values (except where *p* < .001). Model-level statistics include adjusted R^2^ and F-tests. Predictors in the originality models were theory-driven and based on prior findings, while usefulness models were selected using stepwise regression based on AIC. Standardized coefficients were computed using z-scored variables to facilitate effect size comparison across predictors.

**Table 3:** Descriptive statistics of all variables analyzed in Study 2.

Task	Measure	Mean	SD
Relatedness judgment task (RJT)	Clustering coefficient (CC)	0.15	0.14
	Global efficiency (E-global)	29.01	14.24
	Modularity (Q)	0.26	0.18
	Eigenvector centrality	0.20	0.02
	Weighted average degree	18.44	11.77
Creative problem solving (CPS)	Ideation fluency (originality)	2.6	2.45
	Objective originality	2.48	0.622
	Subjective originality	4.14	0.98
	Ideation fluency (Usefulness)	2.5	2.34
	Objective usefulness	2.99	0.41
	Subjective usefulness	4.38	0.66
Alternative uses task (AUT)	Ideation fluency (Originality)	4.11	2.71
	Objective originality	1.75	0.49
	Subjective originality	3.64	1.16
	Ideation fluency (Usefulness)	4.23	2.80
	Objective usefulness	0.19	0.03
	Subjective usefulness	4.3	0.69

*Note*. *N* = 106.

**Table 4:** Summary of regression models predicting creativity scores in the AUT (objective and subjective originality).

Outcome	F	*p*	Adj. R^2^	Predictor	Β	SE	*t*	*p*
Originality score	3.59	.03	0.04	(Intercept)	1.51	0.106	14.25	<.001
				Weighted avg. degree	0.003	0.004	0.94	.34
				Fluency (originality)	0.04	0.01	2.29	.02
Originality judgment	1.74	.16	0.02	(Intercept)	4.48	0.96	4.66	<.001
				Global efficiency	0.01	0.01	1.33	.18
				Eigenvector centrality	−7.38	5.38	−1.37	.17
				Fluency (originality)	0.06	0.04	1.52	.13
Usefulness score	3.24	.02	0.06	(Intercept)	0.15	0.01	9.66	<.001
				Global efficiency	0.001	0.0003	3.11	.002
				Modularity	0.05	0.02	2.05	.04
				Fluency (usefulness)	−0.0004	0.001	−0.36	.72
Usefulness judgment	1.87	.13	0.02	(Intercept)	4.97	1.07	4.61	<.001
				Modularity	0.32	0.67	0.48	.62
				Eigenvector centrality	−5.6	2.47	−2.27	.02
				Fluency (usefulness)	0.01	0.02	0.58	.55

*Note*—Reported values include unstandardized regression coefficients (B), standard errors (SE), *t*-values, and exact *p*-values (unless *p* < .001). Model-level statistics include adjusted R^2^ and F-tests. Predictors for both originality and usefulness models were selected based on theoretical rationale and prior findings from Studies 1 and 2. No stepwise selection was used. All predictors were entered simultaneously without standardization.

**Table 5:** Summary of regression models predicting creativity scores in the CPS task (objective and subjective originality and usefulness).

Outcome	F	*p*	Adj. R^2^	Predictor	Β	SE	*t*	*p*
Originality score	0.22	.79	−0.01	(Intercept)	2.4	0.12	18.87	<.001
				Weighted avg. degree	0.003	0.005	0.61	.54
				Fluency (originality)	0.005	0.02	0.22	.82
Originality judgment	0.61	.609	−0.01	(Intercept)	3.21	0.83	3.82	<.001
				Global efficiency	−0.001	0.008	−0.12	.89
				Eigenvector centrality	4.27	4.66	0.91	.36
				Fluency (originality)	0.03	0.03	0.86	.39
Usefulness score	2.28	.08	0.03	(Intercept)	3.43	0.18	18.81	<.001
				Global efficiency	−0.007	0.003	−2.03	.04
				Modularity	−0.75	0.29	−2.51	.01
				Fluency (usefulness)	−0.007	0.01	−0.46	.64
Usefulness judgment	1.22	.29	0.004	(Intercept)	3.47	0.58	5.89	<.001
				Weighted avg. degree	−0.007	0.007	−1.006	.31
				Eigenvector centrality	5.12	3.28	1.56	.12

*Note*—Reported values include unstandardized regression coefficients (B), standard errors (SE), *t*-values, and exact *p*-values (unless *p* < .001). Model-level statistics include adjusted R^2^ and F-tests. All predictors were entered simultaneously based on theoretical considerations and prior results from Studies 1 and 2. No stepwise selection procedure was applied, and all variables were included in their original (unstandardized) form.

## Data Availability

The data and analysis code of both studies can be found at https://doi.org/10.17605/OSF.IO/49TFD (accessed on 1 November 2025).

## Supplementary Materials

The following supporting information can be downloaded at https://www.mdpi.com/article/10.3390/jintelligence14030041/s1, Text S1: Dilemmas presented in the creative problem solving task; Table S1: Wordlist used in the relatedness judgment task.

## Abbreviations

The following abbreviations are used in this manuscript:

AUT	Alternative uses task
CPS	Creative problem solving
DT	Divergent thinking
MAD	Maximum associative distance
OCSAI	Open Creativity Scoring with AI
RJT	Relatedness judgment task
SemNet	Semantic memory network
