# Supplementary material for: Semantic Memory Structure and Self-Evaluation of Creativity: Evidence Across Tasks and Dimensions
Source: J Intell. 2026 Mar 4;14(3):41. doi: 10.3390/jintelligence14030041 (PMC13027982; doi:10.3390/jintelligence14030041)
Supplement: Supplementary file 1 [file jintelligence-14-00041-s001.zip › jintelligence-4008174-supplementary.pdf]

Semantic Memory Structure and Self-Evaluations of Creativity: Evidence Across Tasks and Dimensions

Supplementary Information

Text S1. Dilemmas presented in the creative problem solving task.

Acme

The Engineering Department of Acme Company has been holding wage increases to a 6 percent level. The decision to hold wage increases came about from an effort to reduce product costs: Acme has been forced to raise the price of their product twice in the past year due to increased shipping costs of materials, and upper management does not feel that Acme can remain competitive if there are any future increases in the cost of their product. Unfortunately, the engineering job market in the area stands at about 12 jobs for every one trained engineer. Because of this, "headhunters" are cropping up and are enticing Acme's engineers with "better" jobs and "better" benefits. As of late, turnover among Acme's engineers has increased and productivity has decreased. Also, there is considerable grumbling among current engineers about Acme's policy on wage increases. Upper management feels that much of the dissatisfaction is based upon the headhunters' enticements of better opportunities in other places. The concern at Acme is to maintain a quality group of engineers at a high level of productivity. Upper management at Acme does not know how to solve this problem.

Becky

Becky is a college student who works part-time at Mark's Pizzeria. Mark, the owner of the restaurant, has treated Becky very well. He gave her a job that she needs to help pay her rent when no other business would employ her because she was arrested for shoplifting three years ago. Mark also lets Becky work around her school schedule, and has asked if she wants to be a shift manager in the summers. Becky's roommate Jim also works at the pizzeria, but Jim has been causing a lot of problems at work. He always avoids doing his job, treats customers rudely, and makes a lot of mistakes with orders. Jim recently began stealing food from the pizzeria. Two days ago the pizzeria was short-staffed, so Jim and Becky were the only employees left at closing time. Jim made 10 extra pizzas and took them home to a party he was hosting without paying for them. Becky feels like she needs to do something about Jim's behavior. However, Becky is hesitant to tell Mark about Jim because Jim is a good friend to Becky. Becky also needs Jim to have a job so he can pay his portion of their rent. Becky does not know what to do.

Brian

Brian graduated with an MBA three years ago and has risen through the managerial ranks quickly at a large bank. Approximately a year ago, Brian had a position to fill in his department and hired his college friend's sister, Laura, who had just graduated from college and was in need of a good job. Brian is very close to his college friend Lance, and Lance had highly recommended his sister as a hard worker. In addition, the vice president for human resources at the bank was pleased that Brian had hired a woman for the position since 90% of the employees in Brian's department are men. Laura gets along well with other people in the department, but the quality of her work has been substandard, and she has missed several department meetings. Brian met with Laura and explained what she needed to do to improve

her work, but one month after their discussion, Laura missed another meeting. Brian realizes the importance of recruiting women to work in the department and does not want to upset his friend by firing Laura, but he feels that the poor quality of her work may slow down his career progress. Brian does not know what to do.

Grace

Grace, a talented baker, has been working at Henry's Bakery for a few years now. She is known for her delicious pastries and cakes, and the bakery's customers love her work. However, Grace has been feeling undervalued and underpaid lately. She has been discussing this with her friend Chloe, who works at a different bakery and makes more money than Grace. Chloe has been encouraging Grace to ask for a raise, but Grace is hesitant because she doesn't want to lose her job. The bakery's owner, Henry, has been noticing Grace's talent and has been thinking about offering her a promotion. However, he is also concerned about the current economic situation and the bakery's financial struggles. Henry is not sure what to do.

Joan

In order to increase her job opportunities upon graduation, Joan decided to work as a research assistant with a faculty member in the psychology department during her junior and senior years. Joan was not sure who to work with, so she sought advice from the head of the department, Dr. Johnson. Dr. Johnson suggested that Joan work with his good friend, Dr. Bundt, since Dr. Bundt is well known in his field, has good job contacts, and has many other students working with him. After working with Dr. Bundt for two months, Joan has realized that she is not enjoying the job. The other students working with Dr. Bundt appear to be very happy, but Joan finds that she is not interested in the research project that she was assigned to work on. In addition, she finds that she has a lot of work to do that is very time-consuming,

with very little guidance provided on how to do what is required. Dr. Bundt himself turns out to be unfriendly and difficult to please. Joan is not sure what to do.

Lily

Lily is a waitress at a restaurant that is owned by Ethan. She has been working there for about a year and has a good relationship with Ethan. She is very reliable and always shows up on time. However, she has been having some issues with her boyfriend, Michael, who is also a waiter at the restaurant. They have been having some disagreements about their relationship and it has been affecting her work. Ethan has noticed that Lily has been late a few times and has seemed distracted when working. He is worried that her personal issues may be affecting the restaurant's reputation. Ethan has asked Lily to come to his office to talk about the situation. Lily is hesitant to go because she is not sure what to expect and is worried that Ethan may fire her. She does not know what to do.

Lucas

Lucas operates a small independent bookstore in the downtown area of a mid-sized city. Mia is a frequent customer at the store, and has been browsing through the shelves for over an hour. Alexander, Lucas's business partner, has been feeling the pressure of the recent economic downturn on the book industry. Alexander has been urging Lucas to consider closing the store and finding a more stable job. However, Lucas is hesitant to give up the bookstore, as it has been a passion project for him and he feels a strong connection to the community. Mia has been chatting with Lucas about the store and the current state of the industry, and Lucas is beginning to feel overwhelmed by the conversation. Mia has also been eyeing a specific book on the shelf, and Lucas is unsure of how to handle the situation.

Noah

Noah is an avid gardener who has poured his heart into cultivating a lush vegetable garden. His neighbor Daniel shares his passion, maintaining a breathtakingly beautiful flower garden. The two neighbors frequently exchange gardening tips and seeds. However, their neighbor James recently adopted an energetic puppy that has developed a habit of digging in Noah and Daniel's gardens whenever it escapes the yard. Both Noah and Daniel have kindly discussed the issue with James, but he seems conflicted on how to address the puppy's misbehavior without resorting to harsh punishment. Noah cherishes his friendships with Daniel and James, but the recurring garden destruction is straining those bonds. At the same time, Noah understands James' attachment to his new pet and hesitation to be too strict.

Olivia

Olivia is a college student who works part-time at the campus library. She has been working there for two years and has built a good relationship with her boss, Benjamin. Olivia's best friend, Ava, also works at the library, but Ava has been causing a lot of problems at work. Ava has been neglecting her duties, treating customers rudely, and making a lot of mistakes with the books. Ava recently began stealing books from the library. Two days ago, the library was short-staffed, so Olivia and Ava were the only employees left at closing time. Ava made 10 extra books disappear and took them home to a party she was hosting without paying for them. Olivia feels like she needs to do something about Ava's behavior. However, Olivia is hesitant to tell Benjamin about Ava because Ava is a good friend to Olivia. Olivia also needs Ava to have a job so she can pay her portion of their rent. Olivia does not know what to do.

Sam

Sam is a technician in a large pest control company. Each week, Sam provides pest control assessments and treatments for several dozen regular accounts in his territory and handles any "spot jobs" that come up if he has enough time. Sam enjoys his work because the hours are flexible and he is his own boss. In addition, the pay is commission-based and since Sam is an excellent technician, he makes good money. On the other hand, Sam believes that the work can be dangerous because it calls for lifting and carrying heavy equipment without assistance. Many technicians in the company have had shoulder and back injuries as a result of such strenuous job demands. In the past few months, Sam's family has had additional expenses that Sam's regular work load cannot cover. Sam is working harder and faster, but the depressed economy has made "spot jobs" scarce and has removed any hope of finding a higher paying job elsewhere. Recently, Sam was offered a "spot job" that would pay enough to cover several overdue bills, but would involve a great deal of highly strenuous lifting, carrying and maneuvering of equipment and supplies. Sam needs the money that this job would provide; however, he is afraid of the considerable personal risk. Sam does not know what to do.

Table S1. Word list used in the relatedness judgment task

AUT items	Closest semantic neighbor	SemDis score
belt	conveyor	0.49177
broom	shovel	0.56624
bucket	tub	0.51689
clock	timer	0.54165
knife	blade	0.4521
lamp	bulb	0.432
pencil	crayon	0.39705
pillow	mattress	0.46445
purse	wallet	0.47899
sock	drawer	0.55484
